# Assessing grassland degradation based on abrupt changes in living status of vegetation in a subalpine meadow

**DOI:** 10.3389/fpls.2025.1594772

**Published:** 2025-08-12

**Authors:** Yong Zhang, Yun Zhang, Hasbagan Ganjurjav, Haitao Yue, Kun Tian, Hang Wang, Qiong Zhang, Zijiao Zhao

**Affiliations:** ^1^ Yunnan Key Laboratory of Plateau Wetland Conservation, Restoration and Ecological Services, Southwest Forestry University, Kunming, China; ^2^ Institute of Environment and Sustainable Development in Agriculture, Chinese Academy of Agriculture Sciences, Beijing, China; ^3^ Academy of Forestry Inventory and Planning, National Forestry and Grassland Administration, Beijing, China; ^4^ Yunnan Dashanbao Grus nigricollis National Nature Reserve Management and Protection Bureau, Zhaotong, China

**Keywords:** geo-coding, Mann-Kendall abrupt analysis, threshold, vegetation status, grassland degradation

## Abstract

Grassland degradation impacts and restoration strategies have been extensively studied in existing literature. Nevertheless, current diagnostic approaches for assessing degradation conditions predominantly rely on either empirical or mechanistic approaches, leading to inconsistent findings across studies. Here, we proposed a geo-coding and abrupt analysis based (GAAB) method to identify the degradation conditions of grasslands. The living status of vegetation (*LSV*), which was constructed by cover, height, aboveground biomass, species richness, and the Pielou index of the plant community, served as the indicator in the GAAB method for diagnosing the thresholds of grassland degradation. We developed a rule system to identify abrupt changes in *LSV*. Furthermore, we applied this method in the Dashanbao National Nature Reserve in China as a case study. We found that the subalpine meadows in the Dashanbao National Nature Reserve could be classified into four relative degradation levels, i.e. healthy, light degradation (LD), moderate degradation (MD), and severe degradation (SD), according to the thresholds that identified by abrupt alterations of the *LSV*. The appearance of plant communities, including cover, height, and aboveground biomass, demonstrated a linear decline across the degradation gradient (*p* < 0.05). In contrast, changes in species diversity aligned with the theory of moderate interference, where species richness and the Pielou index were highest in the MD level (*p* < 0.05). Furthermore, the composition of plant communities exhibited a gradual shift from healthy to SD (*p* < 0.05). Our results suggest that the GAAB method could offer a non-empirical approach for diagnosing degradation conditions, thereby enhancing the understanding of the complexities associated with grassland degradation.

## 1 Introduction

Grasslands, which occupy 25% of the world’s total land area and 66.7% of agricultural land (FAO, 2023), play a crucial role in global food supply (Boval and Dixon, 2012). Additionally, grasslands fulfill important ecological functions, including biodiversity maintenance, water supply, soil conservation, and climate regulation (Bengtsson et al., 2019). However, nearly 50% of global grasslands have experienced some degree of degradation due to global changes (Bardgett et al., 2021). Recent studies have increasingly focused on grassland degradation in alpine regions, particularly in the Third Pole, i.e., the Qinghai-Tibetan Plateau. Evidence suggests that climate change and inappropriate anthropogenic activities have led to extensive (exceeding 50%) grassland degradation in this alpine region (Harris, 2010; Cao et al., 2019). Such degradation has severely compromised the ecosystem serves provided by grasslands (Xu et al., 2021; Wang et al., 2022). Consequently, various efforts to reverse this adverse trend have been undertaken (Fu et al., 2023).

Restoration measures for degraded grasslands include grazing exclusion using fences, fertilization, irrigation, and revegetation (Li et al., 2023; Lyons et al., 2023; Ji et al., 2024). However, the restoration of degraded grassland ecosystems may yield high costs and low benefits if restoration measures do not align with the specific degradation conditions (Dong et al., 2023). This misalignment appears to be a pervasive issue. On one hand, current methods for diagnosing grassland degradation are often empirical, relying on qualitative assessments or assumed degradation thresholds (Yao et al., 2016; Zhu et al., 2023), or mechanistic approaches that classify grassland ecosystems into degradation types based on equidistant divisions of plant indicators (Wen et al., 2010; Zhang et al., 2025). Such cursory methods can lead to inaccuracies in classifying degradation levels and may fail to identify restoration factors pertinent to specific grassland ecosystems. On the other hand, the prevailing definition of grassland degradation tends to agricultural productivity-oriented, utilizing net primary productivity-related indices (e.g., the normalized difference vegetation index and plant community cover) to evaluate degradation conditions (Hilker et al., 2014; Wang et al., 2019, 2022). However, recent restoration practices have increasingly emphasized the multiple ecological functions of grassland ecosystems, such as biodiversity maintenance and carbon storage (Hu, 2023). This shift further exacerbates the mismatch between restoration measures and the degradation conditions, potentially hindering effective restoration efforts. Therefore, there is a pressing need to explore accurate, field data-based approaches that consider multiple ecological functions for diagnosing degradation in grassland ecosystems.

One of the most evident performances of grassland ecosystem degradation is the deterioration of the living status of vegetation (*LSV*). Specifically, the cover, height, species richness, and biomass of plant communities, which strongly contribute to the *LSV*, diminish as grassland ecosystems degrade (Wang et al., 2014; Tang et al., 2015; Zhang et al., 2018; Dong et al., 2023). These indices are extensively investigated in field work, as they not only reflect the multifunctional nature of grassland ecosystems but also provide cornerstones for a comprehensive assessment of the *LSV* (Zhang et al., 2015). Identifying the thresholds of *LSV* deterioration using reliable mathematical methods may offer a novel approach for accurately diagnosing the conditions of a grassland ecosystem.

The Mann-Kendall abrupt analysis is commonly employed to detect change points in meteorological factors (e.g., temperature and precipitation) (Liu et al., 2008; Zhao et al., 2008; Xia et al., 2023), runoff (Zhang et al., 2014), and epidemic transmission (Chen et al., 2022). For data series to be suitable for Mann-Kendall abrupt analysis, they must be independent (i.e., each data point should be random) and continuous (i.e., the data series should follow a specific order, such as a chronological order) (Mann, 1945; Kendall, 1949; Liu et al., 2008). In field studies, data from plant communities (i.e., cover, height, species richness, and biomass) are treated as independent within a random sampling framework (Schweiger et al., 2015; Henrys et al., 2024). However, the continuity (or order) of these data is often undefined. Fortunately, a geo-coding technique can establish continuity based on the coordinates of the sampling plots.

This study, using subalpine meadows in the Dashanbao National Nature Reserve in China as a case study, aims to align field-collected data on plant communities with the requirements for Mann-Kendall abrupt analysis through the application of a geo-coding method. Subsequently, a rule system is proposed to identify abrupt changes in *LSV*, facilitating the assessment of degradation conditions in grassland ecosystems.

## 2 Materials and methods

### 2.1 Study area

**Figure 1:**
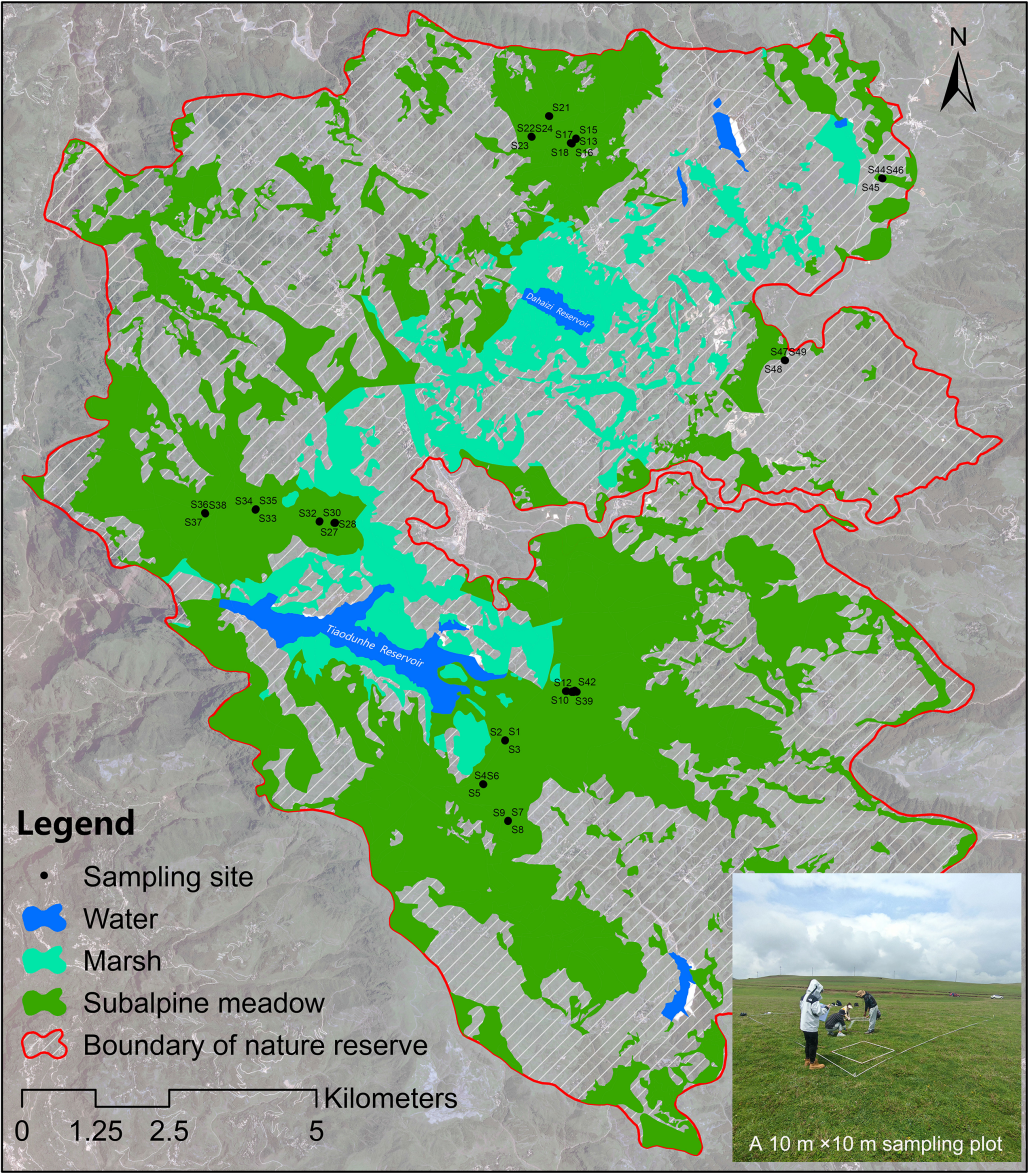
The distribution of sampling sites in Dashanbao National Nature Reserve.

The Dashanbao National Nature Reserve (103°14′55″~103°23′49″ E, 27°18′38″~27°29′15″ N) designated as a Wetlands of International Importance in 2004, serves as a critical wintering habitat for approximately 1,400 individuals of *Grus nigricollis* in China. Subalpine meadows comprise over one-third of the total area of the Dashanbao National Nature Reserve, predominantly located around the marshes within the reserve (**Figure 1**). Consequently, the condition of these subalpine meadows significantly influences both the quality and quantity of habitat available for *G. nigricollis*. These subalpine meadows have experienced degradation due to water erosion and livestock grazing. In response, the management department of the Dashanbao National Nature Reserve has implemented restoration measures, including slope stabilization and plant reseeding, to restore these degraded meadows.

### 2.2 Field investigation and community data handling

Field investigations of subalpine meadows in the Dashanbao National Nature Reserve, Yunnan Province, were conducted in August 2022. A total of 16 randomly established sampling plots measuring 10 m ×10 m was selected in accessible areas of the subalpine meadows (**Figure 1**). In 15 of these plots, three 1 m ×1 m quadrats were positioned along the diagonal, while an additional quadrat was included outside the diagonal in one plot to account for community variation. Within each quadrat, we recorded the species list, cover percentage (%), and average height (cm, measured eight times) of the plant community, as well as the cover (%) and average height (cm, measured five times) for each individual species. Following the investigation, the aboveground biomass of the plant community was collected.

The importance value (*IV*) of each species was calculated based on its relative cover (*RC*) and relative height (*RH*) within the community, specifically in each 1 m ×1 m quadrat. The calculation is as follows:


IV=(RC+RH)/2

where *RC*=*C_i_*/*TC*, *RH*=*H_i_*/*TH*, *C_i_* is the cover of species *i*, *TC* is the summed cover of all species in the plant community, *H_i_* is the average height of species *i*, *TH* is the summed height of all species in the plant community. The total *IV* of all species within a community sum to 1, with the species IV indicating its dominance in the community.

The species *IV* was utilized to examine differences in plant communities across various degradation conditions, specifically focusing on the *β* diversity of plant communities.

### 2.3 The construction of LSV

The *LSV*, used to identify degradation thresholds of grasslands, was constructed by the cover, height, aboveground biomass, species richness, and the Pielou index of plant communities. The construction of the *LSV* involved three steps (Zhang et al., 2015). First, the indices were standardized using the formula: *V_i_′* =*V_i_*/*V_max_*, where *V_i_* ′ presents the standardized value, *V_i_* is the observed value, and *V_max_* is the maximum value of the index. Second, radar maps were generated based on the standardized indices. Each radar map was composed of five triangles that share a common vertex at the coordinate origin. Finally, the area of the radar map was calculated by the formula of $\sum_{i=1}^{5}S_{i}$ , where *S_i_* was the area of triangle *i* that computed using the formula of a × b × sin(θ)/2, where a and b were the lengths of two sides of the triangle and the θ was the included angle. The area of the radar map represented the value of *LSV*.

The contribution of each community index (*Con_i_*) to the *LSV* was calculated using the formula: *Con_i_* =*S_i_*/(2×*LSV*), where *S_i_* presented area correlated with index *i* in the radar map (**Supplementary Figure S1**). Additionally, the uneven contribution of these indices to the *LSV* was evaluated using the coefficient of variation (*CV*) at each degradation level.

### 2.4 Identification of the degradation thresholds based on abrupt alterations of LSV

#### 2.4.1 Three steps to get degradation thresholds

**Figure 2:**
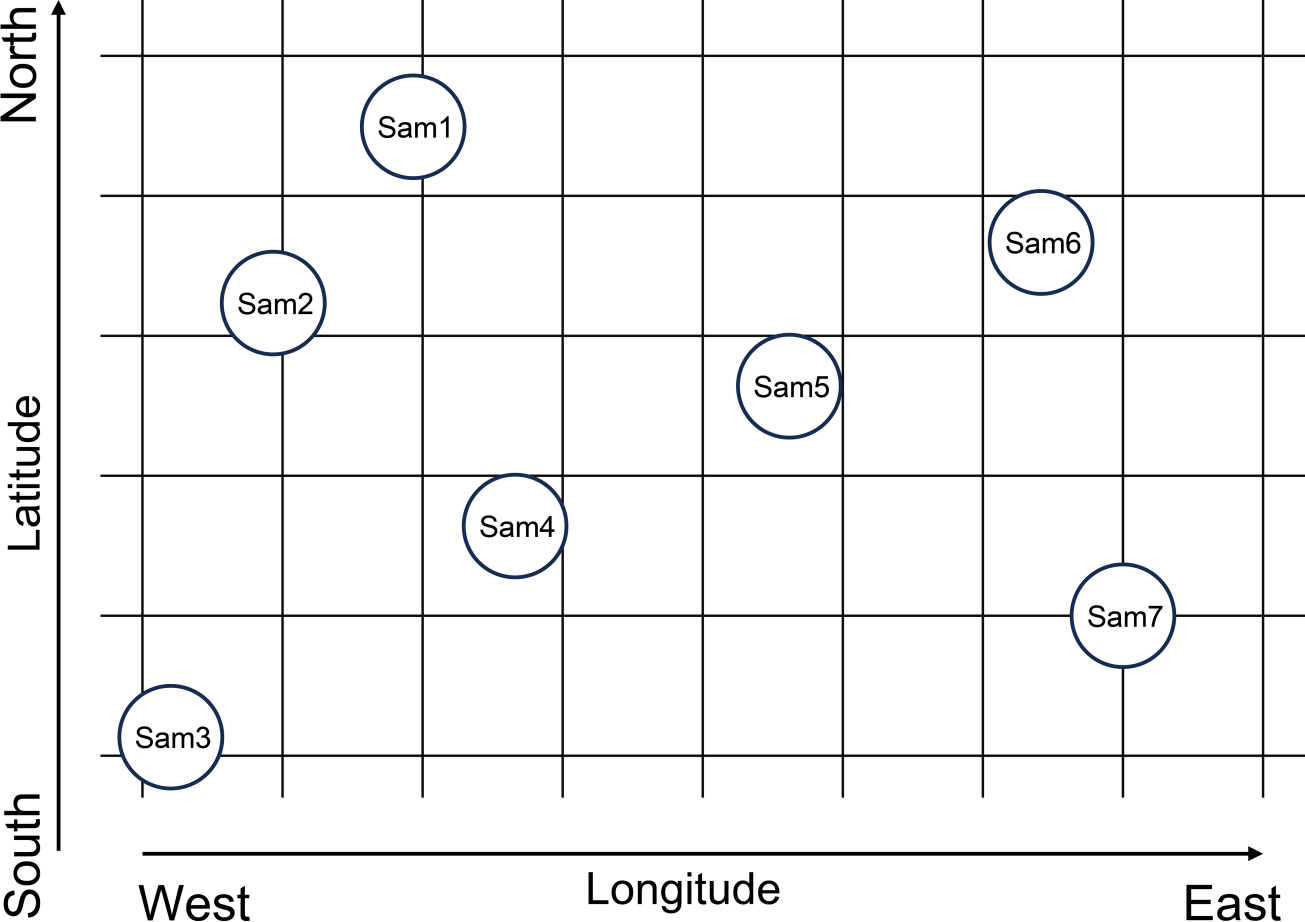
The illustration of geocoding process. Circles with number present sampling sites. Latitude increases from south to north, while longitude increases from west to east.

A geo-coding and abrupt analysis-based (GAAB) method was developed to determine the degradation thresholds of plant communities in this study. The identification of degradation thresholds using the GAAB method involves three steps. First, data sequences were constructed by coding sampling sites according to their longitude and latitude. Coding was performed from four directions (**Figure 2**): from north to south (Sam1, Sam6, Sam2, Sam5, Sam4, Sam7, Sam3) based on descending latitude, from south to north (Sam3, Sam7, Sam4, Sam5, Sam2, Sam6, Sam1) based on ascending latitude, from west to east (Sam3, Sam2, Sam1, Sam4, Sam5, Sam6, Sam7) based on ascending longitude, and from east to west (Sam7, Sam6, Sam5, Sam4, Sam1, Sam2, Sam3) based on descending longitude. This coding approach ensured the stability and accuracy of the identification of thresholds. Second, the Mann-Kendall abrupt analysis, widely employed to detect abrupt changes in climate data (Wang, 2020), was applied to identify abrupt alterations in *LSV* for each coding direction. Comprehensive details regarding the Mann-Kendall abrupt analysis can be found in the literature, with Zhao et al. (2008) providing a thorough outline of the steps and formulas involved. Lastly, degradation thresholds were identified based on the results of the abrupt analysis.

#### 2.4.2 Rules for the identification of degradation thresholds

Several rules must be considered when recognizing degradation thresholds based on the abrupt alterations of *LSV*: (1) Mind the minimum and maximum alterations of the *LSV*. According to our practical experience, limited sample sizes (i.e., less than six) can compromise statistical validity for further analysis. Therefore, both minimum and maximum alteration should be excluded when their respective sampling sites fail to meet the six-sample criteria. (2) Get average value of other alterations of the *LSV*. To obtain the average value of the other alterations in *LSV*, three steps were followed: 1) arrange the abrupt alterations of *LSV* for each coding direction in ascending order; 2) match the abrupt alterations across different directions by rank; and 3) calculate the average value of these abrupt alterations at each rank. And (3) the identified thresholds should be arranged in ascending order, and neighboring thresholds may be averaged if their values are sufficiently close.

### 2.5 Statistical analysis

Variation in *LSV*, cover, average height, species richness, Pielou index, and aboveground biomass of plant communities across different degradation levels was analyzed using a One-way ANOVA, with Tukey’s *post hoc* test applied (*α*=0.05). These statistical analyses were performed in R 4.1.1 using the ‘stats’ package.

To assess variations among plant communities, Principal Coordinates Analysis (PCoA) and Permutational Multivariate Analysis of Variance (PerMANOVA) were conducted using the ‘vegan’ package in R 4.1.1. Specifically, *post hoc* tests were employed to identify differences in plant communities between each pair of degradation gradients, utilizing the ‘pairwiseAdonis’ package in R 4.1.1. Additionally, a Venn diagram was created to explore the distribution of species numbers across degradation gradients, using the ‘VennDiagram’ package in R4.1.1.

## 3 Results

### 3.1 Thresholds of grassland degradation in Dashanbao National Nature Reserve

**Figure 3:**
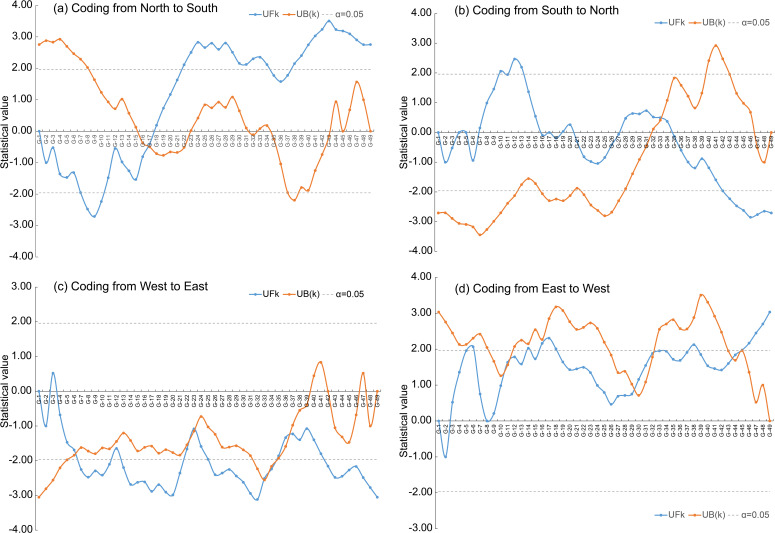
The Mann-Kendall abruption analysis was applied to the LSV (living status of vegetation) to obtain degradation thresholds from four coding directions: north to south **(a)**, south to north **(b)**, west to east **(c)** and east to west **(d)**. For a specified spatial coding direction (e.g., north-to-south), the forward sequence statistic (i.e., UF_k_, blue curve) was computed from the cumulative rank of *LSV* values arranged in an ascending north-to-south sequence, whereas the backward sequence statistic (i.e., UB_(k)_, orange curve) was derived from the cumulative rank of *LSV* values arranged in a descending south-to-north sequence according to Zhao et al. (2008). Significant abrupt change points were identified at the intersections of UF_k_ and UB_(k)_ curves that exceeded the 95% confidence interval bounds (grey dashed lines).

Abrupt alterations in *LSV* were detected when plant samples were coded from north to south and from south to north, with corresponding *LSV* values of 0.76 for both directions (**Figures 3A, B**, **Table 1**). When coding from west to east, five abrupt alterations in *LSV* were identified, with corresponding values of 0.75, 0.91, 0.93, 1.07, and 1.11, respectively (**Figure 3C**, **Table 1**). Similarly, coding from east to west also revealed five abrupt alterations, with *LSV* values of 0.75, 0.87, 0.89, 1.04, and 1.05 (**Figure 3D**, **Table 1**).

**Table 1:** The *LSV* value at different abrupt alterations (AAs).

Coding direction	AAs 1	AAs 2	AAs 3	AAs 4	AAs 5
North→South	0.76				
South→North	0.76				
West→East	0.75	0.91	0.93	1.07	1.11^§^
East→West	0.75	0.87	0.89	1.04	1.05
Average	0.75	0.89	0.91	1.05	1.05

^§^: this alteration should be deleted when calculating the average of AAs 5 because the number of sample sites, whose *LSV* was over 1.11, was less than 6 (**Supplementary Table S1**).

Based on the abrupt alterations in *LSV* detected from the four directions, the degradation thresholds of the grasslands were identified as 0.75, 0.90 (averaged from AAs 2 and AAs 3), and 1.05 (averaged from AAs 4 and AAs 5). Utilizing these thresholds, the grasslands in the Dashanbao National Nature Reserve can be classified into four relative degradation levels: healthy (*LSV* ≥ 1.05), light degradation (LD) (0.90 ≤ *LSV* < 1.05), moderate degradation (MD) (0.75 ≤ *LSV* < 0.90), and severe degradation (SD) (*LSV* < 0.75).

### 3.2 Changes in the appearance of plant communities across degradation levels

The *LSV* exhibited a significant decline across degradation gradients, ranging from 1.27 ± 0.06 in healthy grasslands to 0.51 ± 0.03 in SD grasslands (**Table 2**). Correspondingly, the cover, height, and aboveground biomass of plant communities demonstrated a linear decrease along this gradient. In contrast, species richness and the Pielou index of plant communities displayed a parabolic trend, with the highest value observed in MD grasslands (**Table 2**).

**Table 2:** Plant community indices at different meadow degradation levels (DL), i.e., healthy, light degradation (LD), moderate degradation (MD) and severe degradation (SD) in the Dashanbao National Nature Reserve.

DL	Cover/%	Height/cm	Aboveground biomass/g·m^2^	Species richness	Pielou index	*LSV*
Healthy	95.7 ± 2.9a	26.1 ± 4.1a	318.7 ± 27.6a	17.8 ± 2.7ab	2.20 ± 0.22a	1.27 ± 0.06a
LD	93.6 ± 2.2a	17.8 ± 1.6b	239.2 ± 20.8b	20.0 ± 1.9ab	2.02 ± 0.10ab	0.96 ± 0.02b
MD	89.6 ± 3.1ab	13.6 ± 1.6bc	144.4 ± 9.4c	22.5 ± 1.2a	2.26 ± 0.05a	0.79 ± 0.01c
SD	85.8 ± 1.9b	10.4 ± 1.1c	109.1 ± 9.1c	16.9 ± 0.9b	1.82 ± 0.06b	0.51 ± 0.03d

**Figure 4:**
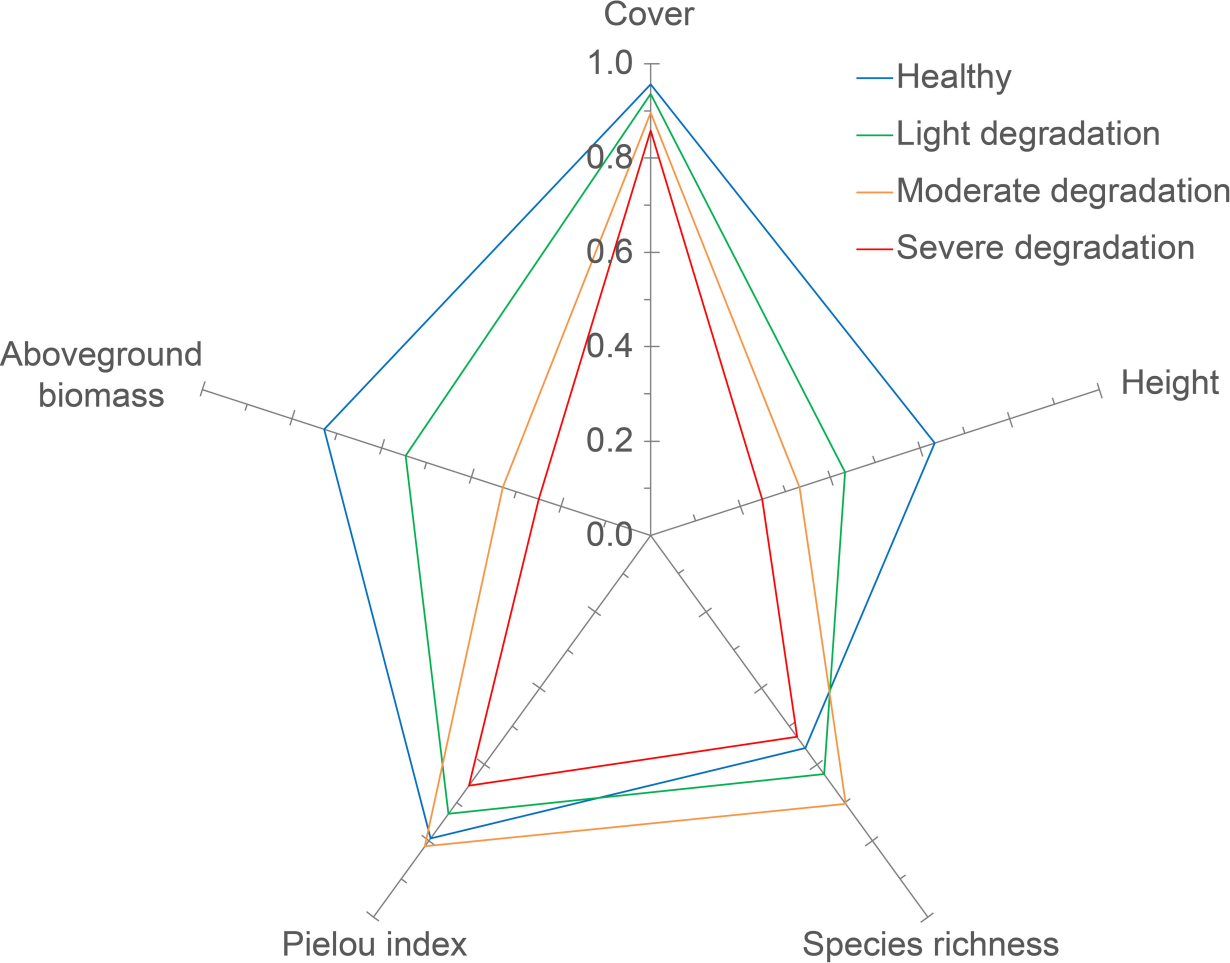
The contribution of each community index to *LSV* at different degradation levels. The radar map for each degradation level was constructed using average values of plant indices. And The contribution of each plant indicator to *LSV* is measured by the proportion of the graphic area associated with that indicator in the total radar chart area.

The contributions of cover, height, species richness, Pielou index, and aboveground biomass of plant communities to *LSV* varied across degradation levels. Notably, the contributions of these indices appeared to be more evenly distributed in LD and SD grasslands compared to MD and healthy grasslands (**Table 3**). Furthermore, the contributions of cover, height, and aboveground biomass of plant communities decreased from 66.0% to 53.8%, while the contributions of *α* diversity indices (i.e., species richness and Pielou index) increased from 34.0% to 46.2% along the degradation gradient (**Figure 4**, **Table 3**). Specifically, species richness contributed the least to *LSV* in healthy grasslands, while plant height was the least contributing index in LD, MD, and SD grasslands (**Figure 4**, **Table 3**).

**Table 3:** Percentage contribution rates (%) of plant community indices to *LSV* at different degradation levels (DL), i.e., health, light degradation (LD), moderate degradation (MD) and severe degradation (SD) in Dashanbao National Nature Reserve.

DL	Cover	Height	Aboveground biomass	Species richness	Pielou index	C.V. ^§^
Healthy	24.3	17.9	23.8	14.9	19.1	0.20
LD	22.5	16.6	22.3	17.8	20.9	0.13
MD	17.8	15.9	16.9	24.2	25.2	0.22
SD	20.0	16.1	17.7	22.3	23.9	0.16
Average	21.2	16.6	20.2	19.8	22.2	0.11

**^§^**: C.V. presents coefficient of variation, which is calculated as the ratio of standard deviation to mean within each individual data row. The C.V. was used to evaluate the uneven contributions of plant indices to *LSV* at each degradation level.

### 3.3 Changes in the composition of plant communities across degradation levels

**Figure 5:**
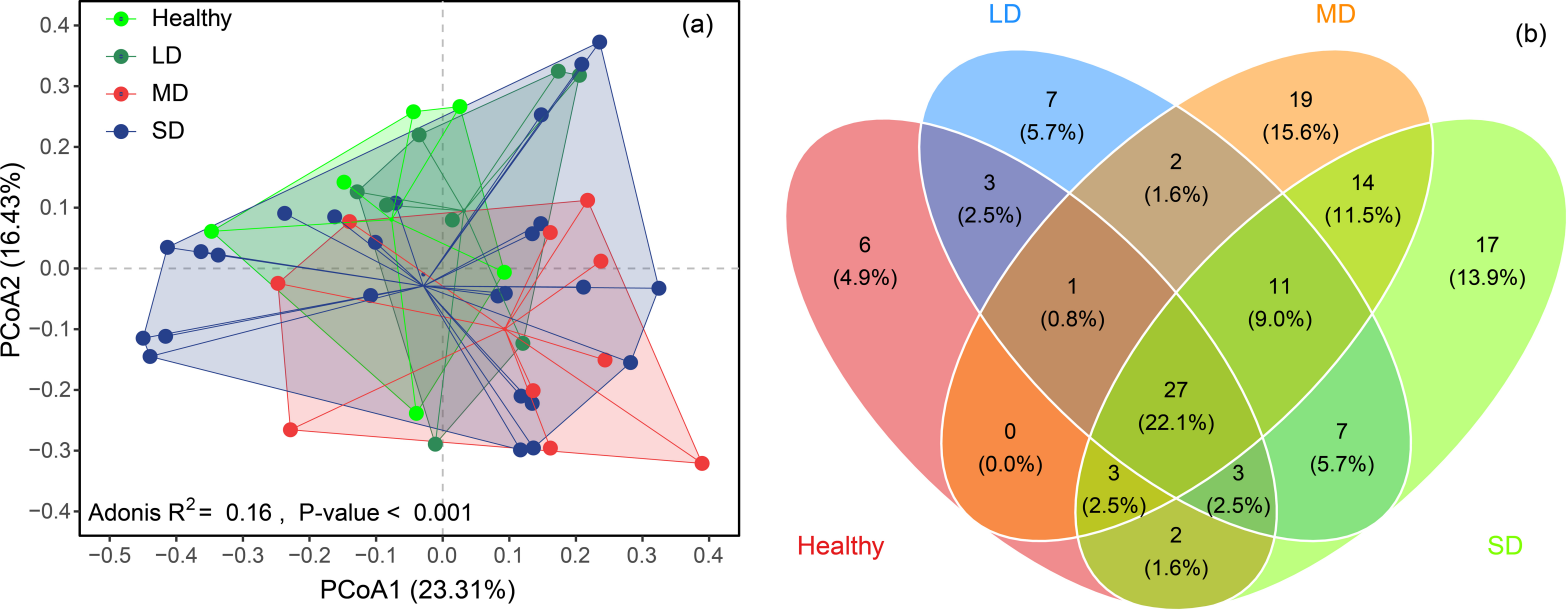
The difference in plant communities among different meadow degradation types, i.e., healthy, light degradation (LD), moderate degradation (MD) and severe degradation (SD) in Dashanbao National Nature Reserve **(a)**. And the number of species distribution among these degradation gradients **(b)**.

The composition of plant communities exhibited gradual variation among degradation gradients (**Figure 5A**). The PCoA revealed that the distribution centers of samples from each degradation level were distinctly separable (**Figure 5A**). *Post hoc* tests indicated that the compositions of plant communities were similar between healthy grasslands and LD grasslands, as well as between LD and MD grasslands. However, significant differences were observed in the composition of plant communities between healthy and MD grasslands, healthy and SD grasslands, LD and SD grasslands, and MD and SD grasslands, respectively (**Table 4**).

**Table 4:** The variations of the composition of plant communities between each two degradation levels.

Pairs	F	R^2^	P
Healthy vs LD	2.09	0.15	0.384
Healthy vs MD	4.12	0.23	**0.006**
Healthy vs SD	3.53	0.11	**0.006**
LD vs MD	1.55	0.09	0.852
LD vs SD	3.17	0.09	**0.030**
MD vs SD	2.63	0.07	**0.024**

Significant differences are highlighted in bold. LD, light degradation; MD, moderate degradation; SD, severe degradation.

The total number of species observed in healthy, LD, MD, and SD grasslands were 45, 61, 77, and 84, respectively. Meanwhile, the number of unique species in each gradient was 6, 7, 19, and 17, respectively (**Figure 5B**). Further analysis revealed that the dominant and main accompanying plant species varied across degradation gradients. *Dactylis glomerata* was identified as the dominant species in healthy grasslands, while *Potentilla lineata* became the dominant species in LD, MD, and SD grasslands (**Table 5**). The accompanying species in healthy grasslands included *P. lineata*, *Rumex acetosella*, *Elymus sinicus*, *Trifolium repens*, and *Festuca ovina*. Most species observed in healthy grasslands transitioned to accompanying species in LD, MD, and SD grasslands. Additionally, *Eragrostis nigra* emerged as another important accompanying species in these degraded grasslands (**Table 5**).

**Table 5:** The dominant and concomitant plant species at different meadow degradation types (DT), i.e., healthy, light degradation (LD), moderate degradation (MD) and severe degradation (SD) in Dashanbao National Nature Reserve.

DT	Top 3 species	Other concomitant species
Healthy	*Dactylis glomerata* (0.14 ± 0.06) *Potentilla lineata* (0.09 ± 0.02) *Rumex acetosella* (0.09 ± 0.02)	*Elymus sinicus* (0.04 ± 0.03) *Trifolium repens* (0.04 ± 0.02) *Festuca ovina* (0.04 ± 0.02)
LD	*Potentilla lineata* (0.17 ± 0.04) *Eragrostis nigra* (0.14 ± 0.04) *Rumex acetosella* (0.09 ± 0.03)	*Festuca leptopogon* (0.06 ± 0.02) *Agrostis clavata* (0.05 ± 0.02) *Dactylis glomerata* (0.04 ± 0.01)
MD	*Potentilla lineata* (0.13 ± 0.02) *Eragrostis nigra* (0.10 ± 0.03) *Festuca ovina* (0.07 ± 0.02)	*Dactylis glomerata* (0.06 ± 0.03) *Trifolium repens* (0.05 ± 0.02) *Festuca leptopogon* (0.05 ± 0.02)
SD	*Potentilla lineata* (0.16 ± 0.02) *Eragrostis nigra* (0.12 ± 0.02) *Dactylis glomerata* (0.08 ± 0.02)	*Trifolium repens* (0.07 ± 0.02) *Rumex acetosella* (0.04 ± 0.02) *Festuca ovina* (0.04 ± 0.01)

The importance value of species (mean ± S.E.), which was calculated as the average of relative cover and relative height of the species, is shown in the bracket.

## 4 Discussion

Grassland degradation is a nonlinear process that typically occurs progressively rather than abruptly, often under stable disturbances such as livestock grazing (Tiscornia et al., 2019; Zhang et al., 2020). An effective diagnostic method for assessing grassland ecosystem degradation should reflect this characteristic. Many studies that classify grassland degradation levels based on empirical or mechanistic approaches emphasize a linear decline in cover, height, and biomass of plant communities (Li et al., 2023), creating a simplistic perception of degradation for stakeholders involved in grassland management. In this study, the subalpine meadows of the Dashanbao National Nature Reserve were classified into four relative degradation levels, i.e., healthy, light degradation, moderate degradation, and severe degradation, using the GAAB method. We observed that changes in the structure of the plant community (including *α* diversity, composition, and dominant species) were nonlinear and progressive, while functional indices (cover, height, and aboveground biomass) exhibited a linear reduction with increasing degradation levels. This asynchronous degradation process between the structural and functional aspects of plant communities underscores the nonlinear nature of grassland degradation. Furthermore, the non-equidistant degradation thresholds of *LSV* reflect a nonlinear degradation process as well. We found that the thresholds changed from 1.27 to 0.96 (*Δ LSV* = 0.31) from healthy to light degradation, with a *Δ LSV* of 0.17 from light to moderate degradation, and 0.28 from moderate to severe degradation. This indicated that *LSV* deterioration was rapid in the initial and final stages of degradation, while transitioning more smoothly during the intermediate stage. The response of plant communities to environmental disturbances may contribute to this phenomenon. For example, the height and cover of grassland plants declined sharply at first, followed by a gradual reduction during trampling experiments (Cole, 1995; Hill and Pickering, 2009).

The criterion for defining the “healthy status” of a grassland ecosystem is a critical consideration when assessing degradation levels. This “healthy status” should be contextualized by geographical location and types of disturbances, as there is no universal criterion applicable to all grassland ecosystems. Two strategies can be employed to define the “healthy status”. Firstly, it can be established based on historical records or inferred from model simulations, especially in theoretical discussions at larger temporal and spatial scales. For instance, ecologists might predict potential productivity and plant species distribution under varying climatic conditions (Gao et al., 2016; Han et al., 2019), with optimal model outcomes serving as a benchmark for “healthy status”. However, a more pragmatic approach is needed for effective grassland restoration practices. According to the law of distance attenuation, the impact of environmental disturbances on grassland ecosystem diminishes with distance from the disturbance source (Huang et al., 2021). Therefore, minimally disturbed grassland sections can serve as factual benchmarks for defining “healthy status”. By conducting systematic field surveys across the study area, ecologists may delineate these reference conditions that can guide the classification of degradation levels. This “healthy status” can also guide stakeholders in setting restoration targets. In the present study, the “healthy status” of subalpine meadows in the Dashanbao National Nature Reserve was determined by identifying plant communities with the highest *LSV*. We found that metrics such as cover, height, aboveground biomass, species *α* diversity, and dominant species under this “healthy status” consistently outperformed those in other degradation levels. Thus, restoration targets for grasslands in the Dashanbao National Nature Reserve could be established based on the plant indices characteristic of this “healthy status”.

Locking in priority restoration factors can significantly enhance the efficiency of grassland restoration efforts. Within the GAAB method, we can identify key restoration factors based on the contributions of various plant indices to the *LSV*. In this study, we observed a decrease in the contributions of cover, height, and aboveground biomass of plant communities to the *LSV*, while the contributions of *α* diversity indices increased with the degradation levels. In line with the diversity-stability relationship in grassland ecosystems (Tilman et al., 2006), it is crucial to prioritize the enhancement of ecosystem stability by increasing plant *α* diversity, particularly in LD and SD areas within the Dashanbao National Nature Reserve. Furthermore, selecting native plant species, such as *Elymus sinicus* and *Eragrostis nigra*, is essential to mitigate issues related to species invasion during restoration practices. Conversely, in regions experiencing high levels of degradation, the focus should shift toward improving cover and biomass of the plant community. Soil nutrient regulation strategies, such as fertilization, can effectively promote these parameters (Holatko et al., 2023; He et al., 2024; Pittarello et al., 2024). It should be noted that it is imperative to develop a specific restoration plan that outlines the number and abundance of plant species, as well as the type and quantity of fertilizers to be applied in these restoration efforts.

Every ecological method must be applied under specific conditions. Due to the heterogeneous distribution of ecological elements (Scheiner and Willig, 2007; Huang et al., 2023), the GAAB method should be applicable only to the monitoring of a particular grassland ecosystem or to historical assessments of the same grassland ecosystem. It is not suitable for evaluating degradation in grassland ecosystems characterized by high heterogeneity or across diverse types. Additionally, increasing the sample size or the number of observations will undoubtedly help improve the accuracy of the GAAB method. Furthermore, the *LSV*, which integrated plant community attributes such as cover, height, aboveground biomass, species richness, and the Pielou index to detect degradation thresholds in this study, can be flexibly tailored to diverse research contexts (Ganjurjav et al., 2022). In this study, all plant indices were assigned equal weights during the construction of the *LSV*, as determining the relative importance of individual indices for characterizing plant community status proved challenging. However, future research could implement differential weighting of standardized indices in *LSV* construction, provided that reliable methods for quantifying their relative ecological significance become available.

## 5 Conclusions

The GAAB method, which utilized the *LSV* as a monitoring index, effectively captured the nonlinear and progressive degradation processes, identifies the “healthy status”, and prioritized restoration factors for the subalpine meadows in the Dashanbao National Nature Reserve. This approach offers a non-empirical framework for diagnosing degradation conditions. The implementation of this novel method could enhance the understanding of the complexities associated with grassland degradation and contribute to the development of practical knowledge for the restoration of degraded grassland ecosystems.

## Data Availability

The raw data supporting the conclusions of this article will be made available by the authors, without undue reservation.
