# Supplementary figures and images for: Assessing grassland degradation based on abrupt changes in living status of vegetation in a subalpine meadow
Source: Front Plant Sci. 2025 Aug 12;16:1594772. doi: 10.3389/fpls.2025.1594772 (PMC12378160; doi:10.3389/fpls.2025.1594772)

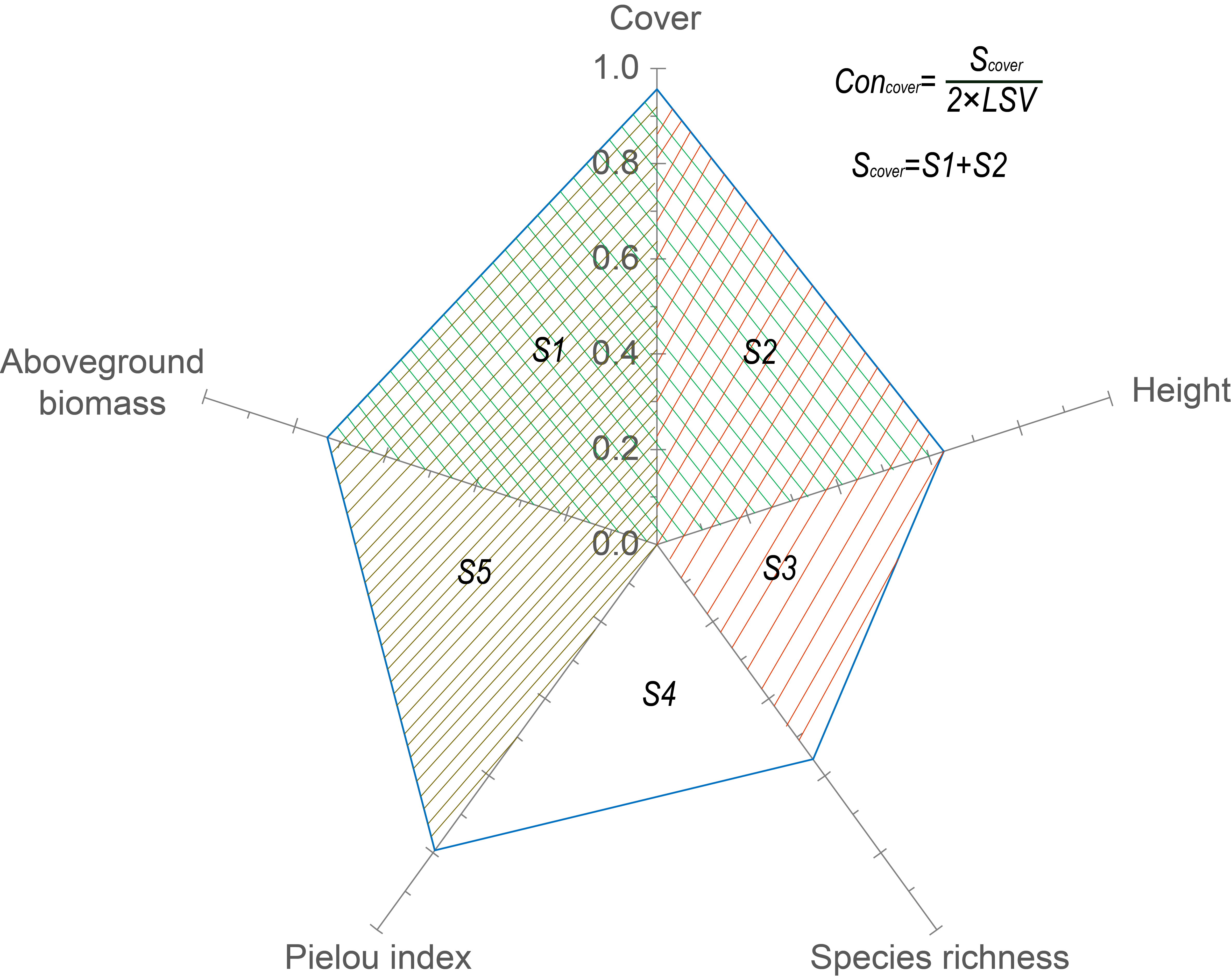

Supplement: Supplementary file 1 [file Image1.jpg]
