# Supplementary material for: Assessing grassland degradation based on abrupt changes in living status of vegetation in a subalpine meadow
Source: Front Plant Sci. 2025 Aug 12;16:1594772. doi: 10.3389/fpls.2025.1594772 (PMC12378160; doi:10.3389/fpls.2025.1594772)
Supplement: Supplementary file 2 [file Table1.docx]

**Table S1** The number of sample sites at each degradation threshold as well as at the minimum and maximum abrupt alterations of the *LSV*

| Groups | *LSV* value | Number of sample sites | Ratio /% |
| --- | --- | --- | --- |
| The first threshold (0.75) | *LSV* < 0.75 | 25 | 51.0 |
| The second threshold (0.90) | 0.75 ≤ *LSV* < 0.90 | 10 | 20.4 |
| The third threshold (1.05) | 0.90 ≤ *LSV* <1.05 | 7 | 14.3 |
|  | 1.05 ≤ *LSV* | 7 | 14.3 |
| The minimum alteration (0.75) | *LSV* < 0.75 | 25 | 51.0 |
| The maximum alteration (1.11) | 1.11 ≤ *LSV* | 5 | 10.2 |

Note: the total number of sample sites was 49, i.e., the total number of sites that are divided by these three thresholds.
